# A more effective CT synthesizer using transformers for cone-beam CT-guided adaptive radiotherapy

**DOI:** 10.3389/fonc.2022.988800

**Published:** 2022-08-25

**Authors:** Xinyuan Chen, Yuxiang Liu, Bining Yang, Ji Zhu, Siqi Yuan, Xuejie Xie, Yueping Liu, Jianrong Dai, Kuo Men

**Affiliations:** ^1^ National Cancer Center/National Clinical Research Center for Cancer/Cancer Hospital, Chinese Academy of Medical Sciences and Peking Union Medical College, Beijing, China; ^2^ National Cancer Center/National Clinical Research Center for Cancer/Hebei Cancer Hospital, Chinese Academy of Medical Sciences, Langfang, China; ^3^ School of Physics and Technology, Wuhan University, Wuhan, China

**Keywords:** adaptive radiotherapy, CBCT, deep learning, transformer, image quality

## Abstract

**Purpose:**

The challenge of cone-beam computed tomography (CBCT) is its low image quality, which limits its application for adaptive radiotherapy (ART). Despite recent substantial improvement in CBCT imaging using the deep learning method, the image quality still needs to be improved for effective ART application. Spurred by the advantages of transformers, which employs multi-head attention mechanisms to capture long-range contextual relations between image pixels, we proposed a novel transformer-based network (called TransCBCT) to generate synthetic CT (sCT) from CBCT. This study aimed to further improve the accuracy and efficiency of ART.

**Materials and methods:**

In this study, 91 patients diagnosed with prostate cancer were enrolled. We constructed a transformer-based hierarchical encoder–decoder structure with skip connection, called TransCBCT. The network also employed several convolutional layers to capture local context. The proposed TransCBCT was trained and validated on 6,144 paired CBCT/deformed CT images from 76 patients and tested on 1,026 paired images from 15 patients. The performance of the proposed TransCBCT was compared with a widely recognized style transferring deep learning method, the cycle-consistent adversarial network (CycleGAN). We evaluated the image quality and clinical value (application in auto-segmentation and dose calculation) for ART need.

**Results:**

TransCBCT had superior performance in generating sCT from CBCT. The mean absolute error of TransCBCT was 28.8 ± 16.7 HU, compared to 66.5 ± 13.2 for raw CBCT, and 34.3 ± 17.3 for CycleGAN. It can preserve the structure of raw CBCT and reduce artifacts. When applied in auto-segmentation, the Dice similarity coefficients of bladder and rectum between auto-segmentation and oncologist manual contours were 0.92 and 0.84 for TransCBCT, respectively, compared to 0.90 and 0.83 for CycleGAN. When applied in dose calculation, the gamma passing rate (1%/1 mm criterion) was 97.5% ± 1.1% for TransCBCT, compared to 96.9% ± 1.8% for CycleGAN.

**Conclusions:**

The proposed TransCBCT can effectively generate sCT for CBCT. It has the potential to improve radiotherapy accuracy.

## 1 Introduction

As a tool for image-guided radiotherapy, cone-beam computed tomography (CBCT) equipped with radiotherapy units can acquire three-dimensional images of patients at the treatment position. The positioning error of fractional treatment can be corrected by registering fractional CBCT with simulation CT images. CBCT imaging can also be applied for adaptive radiation therapy (ART) to ensure the accuracy of dose delivery when the patient’s anatomy changes significantly during the treatment course (1–3). However, the greatest challenge of CBCT is its low image quality, which limits its application for precise radiotherapy (2, 3).

Several conventional methods have been proposed to improve the image quality of CBCT. They are classified into hardware-based and software-based methods. Hardware, such as anti-scatter grid (4) and x-ray beam blocker with a strip pattern (5), are employed to reduce the scatter photons, reducing the imaging system’s quantum efficacy. Meanwhile, additional devices need to be set up on the onboard imagers to use these methods. These problems are not exited in software approaches. Ray tracing (6) and Monte Carlo (7) methods can estimate scatter distribution to correct the CBCT projections. Additionally, iterative reconstruction (8, 9) is implemented to obtain high-quality images from the limited projections. These methods are promising but limited by the huge computational cost.

Recently, deep learning methods, especially the convolutional neural network (CNN)-based model, have been proven promising in image processing due to their advantages in leveraging local context and enabling a large reception field. CNN has been explored to improve the quality of CBCT images by correcting the projections (10–13) and generating synthetic CT (sCT) images (14–16). However, it is limited when substantial anatomical changes exist between CBCT and planning CT due to its supervised learning pattern (17, 18). Since the exactly matched CBCT and CT images are nearly unavailable, some studies employed unsupervised learning to enhance the quality of CBCT images to CT level in the image domain. The generative adversarial network (GAN)-based models, especially the cycle-consistent adversarial network (CycleGAN) (19), are suitable for image transferring with unpaired data. Liang et al. (20) used CycleGAN to preserve the anatomical structure of CBCT and improve its image quality on the head and neck. For the parts with substantial organ dislocations, such as the abdomen, Liu et al. (21) employed a deep-attention CycleGAN and copied the air pockets observed in CBCT and CT to solve the mismatching problem. The cycle-consistent is helpful to the keep the raw structure of the cycle-consistent loss is helpful to the keep the raw structure of CBCT. Kida et al. (22) employed more loss function parts to visually enhance the CBCT images. Uh et al. (23) combined adjacent anatomic data and normalized age-dependent body sizes in children and young adults to improve the training set, and got a better CycleGAN model.

In the past few years, the transformer has been one of the popular architectures for deep learning task, since it can take advantage of modeling long-range dependencies based on the attention mechanism (24, 25). Now, the transformer is expected to handle the more medical image processing tasks. Wu et al. (26) successfully employed Vision Transformer (27) to recognize diabetic retinopathy grade with more accuracy than the CNN-based model. Yang et al. (28) designed a transformer-based deformable image registration network and achieved robust registration and promising generalizability. Zhang et al. (29) employed the transformer blocks for low dose CT denoising and produced superior results. Liu et al. (30) proposed the Swin transformer to implement hierarchical architecture using a non-overlapping shifted windowing scheme to obtain greater efficiency, achieving great progress in image classification, dense prediction, and semantic segmentation.

Inspired by the emerging advantages of transformers, we proposed a novel transformer-based network, called “TransCBCT”, to convert CBCT to sCT. We hypothesized that sCT generated by the proposed TransCBCT can improve image quality CBCT-based image-guided radiotherapy. The clinical value was tested on segmentation and dose calculation, which is significant to radiotherapy. To the best of our knowledge, this is the first attempt to apply transformer in synthesizing CT from CBCT. The experiments have demonstrated that it is superior to the state-of-the-art method (CycleGAN) in improving CBCT. This study may provide a more effective network to the long-standing challenges in the clinical application of CBCT.

## 2 Materials and methods

### 2.1 Data collection

Data of 91 patients with prostate cancer were collected in this study. The planning CT images and daily CBCT images were acquired and registered. The planning CT images were acquired with a CT simulator (SOMATOM Definition AS 40, Siemens or Brilliance CT Big Bore, Philips) with the following parameters: voltage: 120 kV; exposure: 280 (Siemens) or 240 (Philips) mAs; image resolution: 512 × 512; pixel size: 1.27 × 1.27 mm^2^; slice thickness: 3 mm. The CBCT images were scanned on a Varian On-board Imager with the following parameters: voltage: 125 kV; exposure: 1,080 mAs; rotation range: 360°; projections: 900 frames; image resolution: 512 × 512; pixel size: 0.91 × 0.91 mm^2^; slice thickness: 1.91 mm. The radiotherapy was implemented on a Varian Edge radiosurgery system. Deformable registration was implemented using the MIM software (v.7.0.1, MIM Software Inc., Cleveland, OH, USA) to make the planning CT images paired to the CBCT images. The deformed CT images were resampled to have the same spatial resolution and pixel size as the reference CBCT images. The gray value of pixels outside the patient body was set to zero to avoid background influence. Institutional Review Board approval was obtained for this retrospective analysis.

### 2.2 The transformer framework

**Figure 1:**
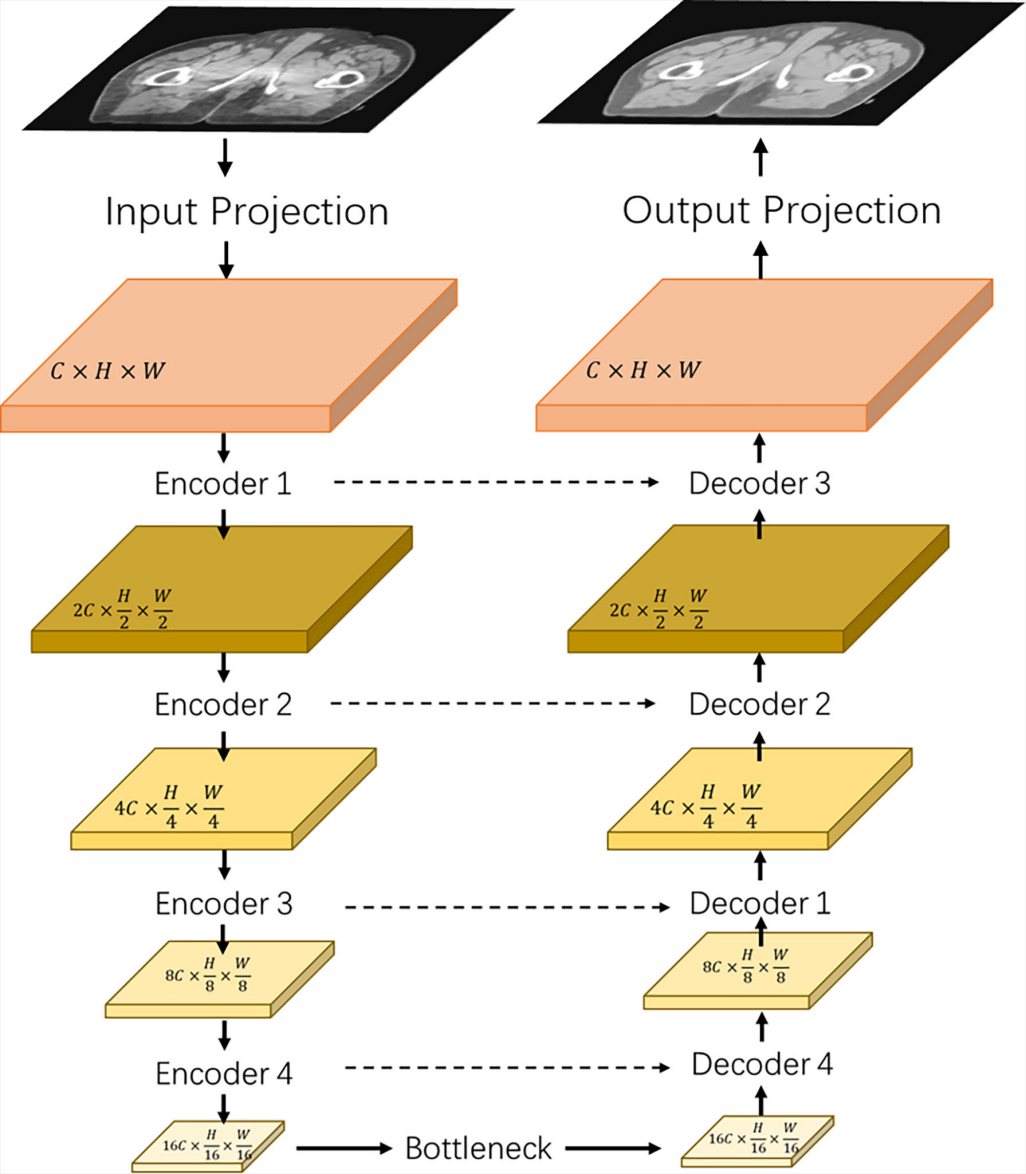
Architecture of the proposed TransCBCT. The network was constructed using a hierarchical encoder–decoder structure with skip connection. The encoder and decoder used a transformer block to construct a hierarchical structure, which was efficient to extract features and recover the image structures and details.

**Figure 1** shows the architecture of the proposed transformer network (named as “TransCBCT”), which was a U-shape hierarchical encoder–decoder structure with skip connection. For the training stages, the input of TransCBCT was a 2D CBCT image, and the output was the corresponding 2D deformed CT image. The transformer blocks were adopted into the encoder and decoder. The main design for the transformer part is hierarchical structure and shifted-window based multi-head self-attention method (SW-MSA). This is helpful to capture global information and save computing source. Instead of using a pure transformer-based structure, some convolution layers were employed to help enhance the local detail.

The input projection adopted a 3 × 3 convolution layer with LeakyReLU to extract shallow feature maps *X* ∈ *ℝ* ^*C*×*H*×*W*^ from the CBCT image *I* ∈ *ℝ* ^1×*H*×*W*^ with *H* and *W* being the height and width of the map. A 3 × 3 convolutional layer was used as the output projection. Four transformer-based encoders and one transformer-based bottleneck were used to extract the CBCT image features. Then, we used four transformer-based decoders with skip-connected feature maps to recover the image details.

**Figure 2:**
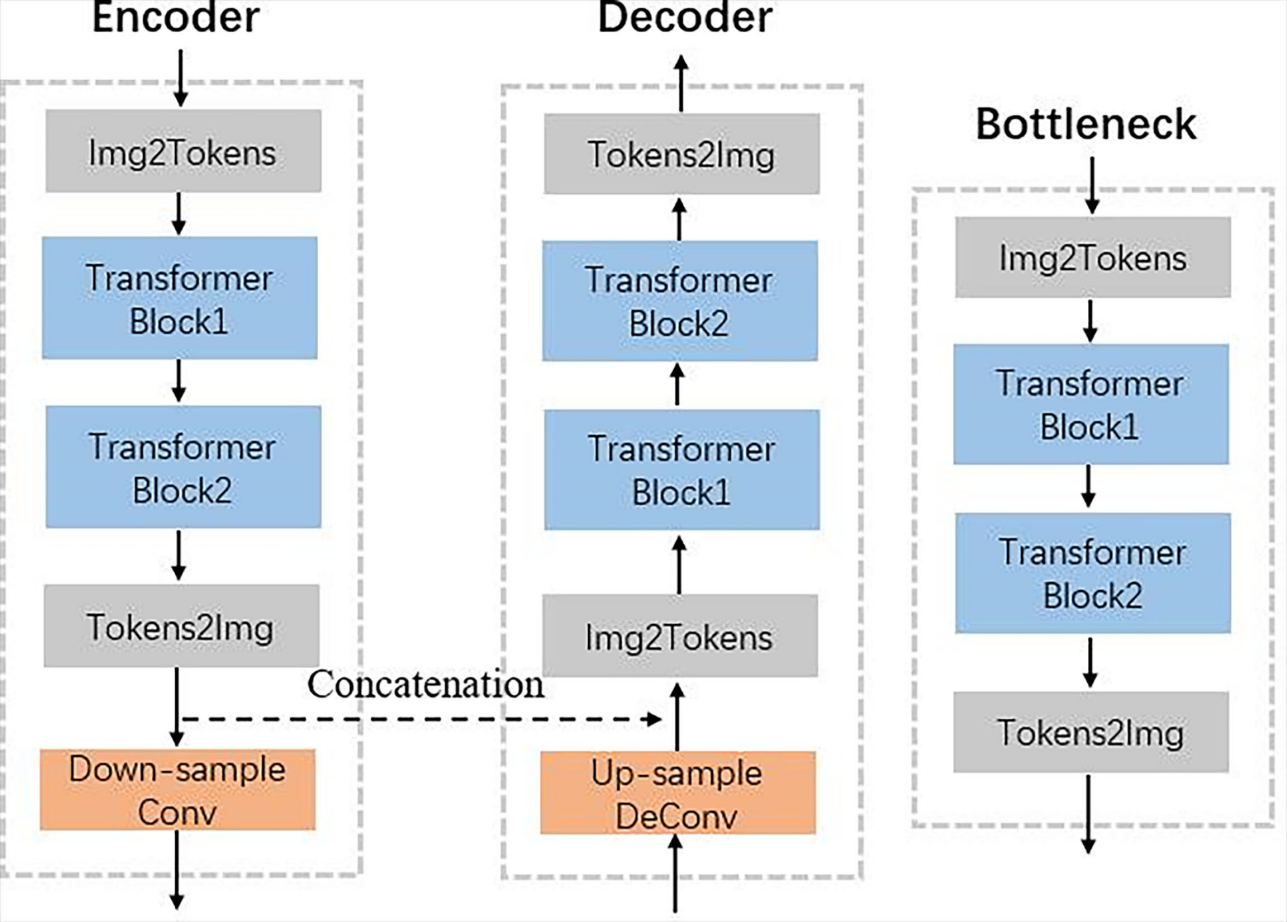
Illustration of encoder, decoder, and bottleneck in the proposed TransCBCT shown in **Figure 1**.

**Figure 2** shows that the hierarchical network used two transformer blocks to capture long-range dependencies in encoder or decoder. For the self-attention calculation, the 2D future maps must be transformed to tokens using the Img2Tokens layer (31). However, we adopted the downsampling and upsampling operators on the 2D feature maps, which need the tokens reshaped to 2D maps by the Tokens2Img layer. For the downsampling, we used a 4 × 4 convolution layer with a stride of 2. Meanwhile, we adopted a 2 × 2 transpose convolution layer with a stride of 2 for the up-sampling.

**Figure 3:**
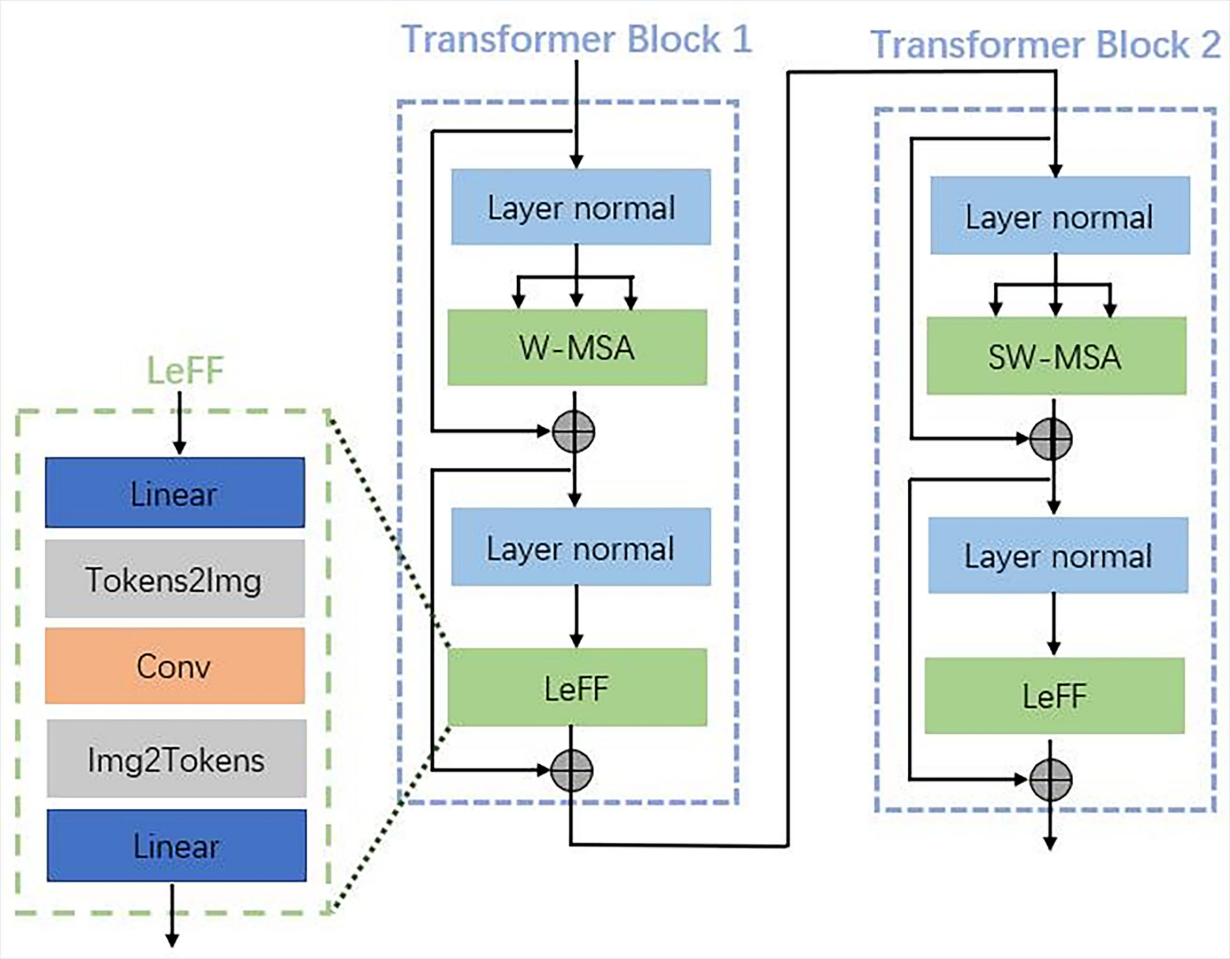
Details of the transformer blocks in the encoder, decoder, and bottleneck shown in **Figure 2**.

Each transformer block consists of two main parts with a normal layer: a multi-header self-attention (MSA) layer and a locally enhanced feedforward network (LeFF) layer, as shown in **Figure 3**. Instead of using global self-attention on the whole image, we implemented self-attention within non-overlapping local windows to reduce the computational cost, the window-based MSA (W-MSA). For the 2D feature maps *X* ∈ *ℝ* ^*C*×*H*×*W*^ with *H* and *W* as the height and width of the maps, we split *X* into non-overlapping windows with a window size of M × M. Then, we obtained the flattened and transposed features *X*_*i*_ ∈ *ℝ* ^*M* ^2^×*C*^ from each window *i*. Next, we performed self-attention on the flattened features in each window. When computing self-attention, the head number is *k*, and the head dimension is *d* = *C*/*k*. We included relative position bias *B* to each head in computing similarity:


(1) $$\text{Attention}\left(Q,K,V\right)=\text{SoftMax}\left(QK/\sqrt{d}+B\right)V$$

where *Q*, *K*, and *V* are the query, key, and value matrices. Moreover, *Q*, *K*, *V*, and *B* are learning parameters. The shift window stage was used to obtain the connection between different windows. As proved in the Swin transformer, shift window-based self-attention was essential to enhance modeling power (30). In transformer block 1, a basal window partitioning scheme was used, and self-attention was conducted in each window. In transformer block 2, the window partitioning was shifted, resulting in new windows. The self-attention computation in the new windows crosses the boundaries of the previous windows in transformer block 1.

To enhance the capability to leverage local context, we employed the LeFF layer that contained a 2D convolutional layer, as shown in **Figure 3**. A linear projection layer was applied to each token to increase its feature dimension. Next, we reshaped the tokens to 2D feature maps and adopted a 3 × 3 depth-wise convolutional layer to capture local information. Then, we flatten back the features to tokens and shrink the channels using another linear layer. The Gaussian Error Linear Unit (GELU) was set as the activation function after each linear/convolutional layer, which is a function that simply multiplies its input by the cumulative density function of the normal distribution at this input.

The loss function contained Charbonnier loss and multiscale structural similarity (MS-SSIM). The Charbonnier loss calibrates the CT numbers as follows:


(2) $$\text{Charbonnier loss}=\sqrt{{\left|\left|y-x\right|\right|}^{2}+e^{2}}$$

where *x* is the ground truth image, *y* is the predicted image, and *e* is a constant, which is 10^−3^.

The MS-SSIM consists of three parts: luminance (L), contrast (C), and structure (S) comparison measures, given as follows:


(3) $$L\left(x,y\right)=\frac{\left(2u_{x}u_{y}+C_{1}\right)}{(u_{x}2+u_{y}2+C_{1})}$$

(4) $$C\left(x,y\right)=\frac{\left(2\sigma_{x}\sigma_{y}+C_{2}\right)}{\left(\sigma_{x}2+\sigma_{y}2+C_{2}\right)}\;$$

(5) $$S\left(x,y\right)=\frac{\left(\sigma_{xy}+C_{3}\right)}{\left(\sigma_{x}\sigma_{y}+C_{3}\right)}$$

where *μ_y_* and *σ_y_* are the https://en.wikipedia.org/wiki/Average and standard deviation of *y*, respectively; *σ_xy_* is the covariance of *x* and *y*; *C*_1_, *C*_2_, and *C*_3_ are constants, set as 1.

The MS-SSIM is defined as follows:


(6) $$\text{MS}-\text{SSIM}\left(x,y\right)={\left[L_{M}\left(x,y\right)\right]}^{\alpha_{M}}\prod_{j=1}^{M}{\left[C_{j}\left(x,y\right)\right]}^{\beta_{j}}{\left[S_{j}\left(x,y\right)\right]}^{\gamma_{j}}$$

where *j* is the image downsampling factor, and *j* = 1 represents the original image.

To quickly restore the image details and calibrate the CT numbers, the Charbonnier loss and MS-SSIM must be added with suitable proportions. Our final result was obtained with a proportion of 9:1 for Charbonnier loss and MS-SSIM, respectively, achieving the best performance in our testing.

### 2.3 Experiments

The paired CT/CBCT images were randomly divided into a training set, a validation set, and a test set. The training set consisted of 4,922 pairs of images from 61 patients, the validation set consisted of 1,222 pairs of images from 15 patients, whereas the test set consisted of 1,206 pairs of images from the remaining 15 patients. Lookahead with a learning rate of 0.001 was set as the optimizer, which is more effective for convergence based on Adam. The CycleGAN was selected for comparation, whose architecture is the same as the study where synthetic kV-CT is generated from megavoltage CT. We chose the “CycleGAN-Resnet” for this work, which contained nine residual blocks in the generator (32). The loss function of CycleGAN contained adversarial loss, cycle-consistent loss, and identity loss (19). In this experiment, all training and testing were conducted on an Nvidia GeForce RTX 3090 GPU.

### 2.4 Evaluation

To validate the proposed TransCBCT, we compared its performance with CycleGAN, which is the state-of-the-art method for CBCT improvement. The evaluation covered image quality of CBCT and its clinical application value: auto-segmentation and dose calculation. The paired *t*-test was performed if the data were normally distributed; otherwise, the Wilcoxon signed-rank test for paired samples non-parametric test was performed. Statistical significance was set at *p* < 0.05.

#### 2.4.1 Image quality

For analyzing the image quality of sCT, the deformable planning CT images were used as the ground truth. The evaluation metrics included mean absolute error (MAE), root mean square error (RMSE), and peak signal-to-noise ratio (PSNR). Their definitions are presented as below:


(7) $$\text{MAE}=\;\frac{1}{\text{N}}\sum_{\text{i}=1}^{\text{N}}\left|{\hat{y}}^{\text{i}}-y^{\text{i}}\right|$$

(8) $$\text{RMSE}=\;\frac{1}{\text{N}}\sqrt{\sum_{\text{i}=1}^{\text{N}}|{\hat{y}}^{\text{i}}-y^{\text{i}}{|}^{2}}$$

(9) $$\text{PSNR}=10\text{log}\frac{{\text{Max}}_{\text{p}}^{\;2}}{\frac{1}{\text{N}}{\sum_{\text{i}=1}^{\text{N}}\left({\hat{y}}^{\text{i}}-y^{\text{i}}\right)}^{2}}$$

where *N* is the number of pixels involved in the calculation; *i* is the *i*th pixel; $\hat{y}$ and y are the test and reference CT number, respectively; and Max_p_ is the possible maximum pixel value in the image.

A higher value of PSNR implies good consistency between sCT and ground truth, while values closer to zero are better for MAE and RMSE. In addition, we select several regions of interest (ROIs) to evaluate the accuracy of CT numbers.

#### 2.4.2 Clinical application value

We focused on two steps of ART workflow: segmentation and dose calculation. Automatic segmentation is often applied in CBCT-based image-guided radiotherapy to improve treatment efficiency. Our previously published network was adopted for segmentation in this study (33). The model is being applied to assist the radiation oncologists in daily clinical work, which have helped them to save time. We chose bladder and rectum for testing, considering their difference between different scan time are difficult to be totally eliminated by the deformed registration. Automatic segmentations were generated on deformed CT, CBCT, sCT (CycleGAN), and sCT (TransCBCT), respectively. To evaluate the accuracy, manual contours on CBCT image were referred to as the ground truth for CBCT, sCT (CycleGAN), and sCT (TransCBCT). The deformed CT were contoured independently since shapes and locations of the organs are different between deformed CT and CBCT. These contours delineated and reviewed by experienced radiation oncologists. The accuracy was compared with metrics of the dice similarity coefficient **(**DSC) and mean distance to agreement (MDA). Higher DSC and lower MDA values indicate better consistency between the automatic segmentations and ground truth. It is worth noting that the model is trained on the planning CT; thus, theoretically, it would get best result on the deformed CT.

As for dose calculations, the Pinnacle treatment planning system (Philips Medical Systems, Fitchburg, WI) was used to create volumetric-modulated arc therapy (VMAT) plans for the 15 patients in the test set. These plans were imported onto different CT images to calculate the dose distribution with collapsed cone convolution algorithm. The dose distribution on deformed CT was regarded as the ground truth of each plan. Then, the dose distributions on the CBCT, sCT (CycleGAN), and sCT (TransCBCT) images were compared to the ground truth using global 3D gamma analysis. In the gamma analysis, a threshold dose value was set to 10% of the prescription dose, and the dose difference criterion was defined as percentage of the prescription dose.

## 3 Results

### 3.1 Image quality

The MAE (HU), RMSE (HU), and PSNR (dB) values of the original CBCT were 66.5 ± 13.2, 90.5 ± 28.3, and 33.4 ± 2.0, respectively. Both CycleGAN and the proposed TransCBCT improved the image quality, with the following metric: MAE, 34.3 ± 17.3 (CycleGAN) vs. 28.8 ± 16.7 (TransCBCT), *p* < 0.05; RMSE, 63.2 ± 36.5 (CycleGAN) vs. 57.0 ± 34.5 (TransCBCT), *p* < 0.05; PSNR, 37.0 ± 2.8 (CycleGAN) vs. 38.0 ± 3.3 (TransCBCT), *p* < 0.05, respectively. Compared with CBCT, the TransCBCT and CycleGAN reduced the MAE value relatively by 56.7% and 48.4%, reduced the RMSE value relatively by 33.6% and 30.1%, and improved PSNR relatively by 13.8% and 10.7%, respectively. The results demonstrate that the overall performance of the proposed TransCBCT was better than the CycleGAN (*p* < 0.05 in MAE, RMSE, and PSNR).

**Figure 4:**
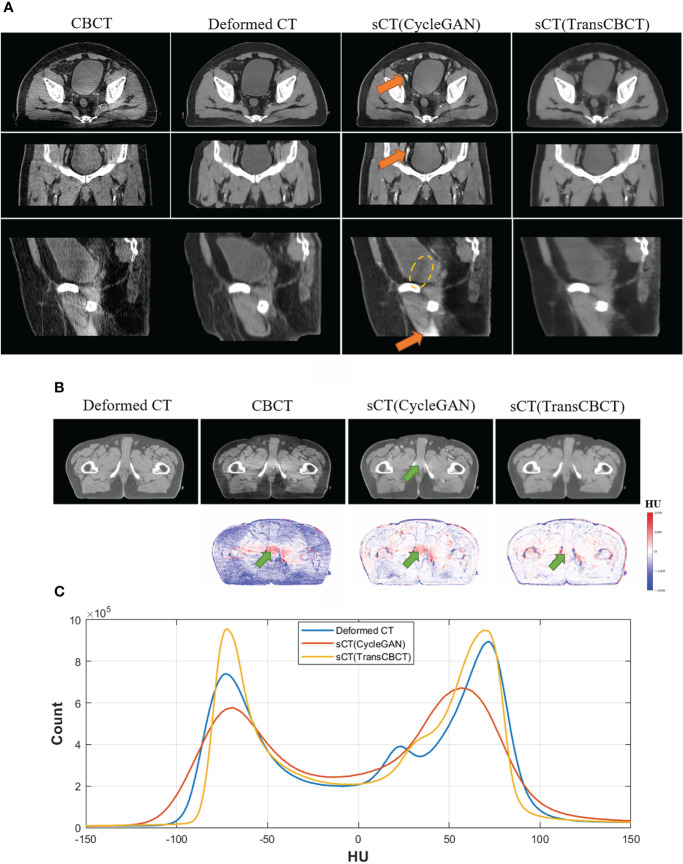
**(A)** Axial, sagittal, and coronal views of one patient. The orange arrows indicate the abnormal structure on sCT images. Yellow lines indicate the obvious beam hardening artifacts. Anatomical differences can be obtained, especially on the bladder sizes and locations, because of the time intervals between CT and CBCT scanning. The TransCBCT and CycleGAN maintained the same structure as CBCT with a clear organ edge. **(B)** Example of an axial slice of the deformed CT, CBCT, and sCT with the HU difference compared to the deformed CT image. The green arrows indicate the “photon starving” artifacts. **(C)** The HU histogram plot calculated on the entire test set.

**Figure 4A** shows an example of a representative patient, which indicates that sCT is more suitable for contouring than the deformed CT image. **Figure 4B** shows a slice of the deformed CT, CBCT, sCT (CycleGAN), and sCT (TransCBCT), with their differences from the ground truth. Both deep learning methods can improve image quality, but the proposed TransCBCT outperforms the CycleGAN, especially in reducing artifacts. As shown by the green arrows, the “photon starving” artifacts are lighter on sCT (TransCBCT) than on CBCT and sCT (CycleGAN). **Figure 4C** shows the HU histogram plots of the entire testing set. Compared to CycleGAN, the proposed TransCBCT improved the HU accuracy corresponding to the deformed CT image.

As indicated by the orange arrow, there were “calcification-like” structures on sCT (CycleGAN), which should not have been observed there. Although Cycle-GANs can be trained on unpaired images due to the cycle-consistent loss, structure preservation remains an issue as input images undergo ambiguous geometric transformations between domains because the models are under-constrained. Meanwhile, TransCBCT did not generate such abnormal structures on sCT images. Besides the “photon starving” artifacts, TransCBCT achieved good performance in reducing the beam hardening effect as marked by the yellow lines. The beam hardening artifacts would affect the accuracy of CT numbers. The CycleGAN only transferred the image style of the deformed CT to CBCT and preserved all structures of CBCT, including the artifact part. Meanwhile, TransCBCT can effectively reduce these artifacts. The results of the ROI test are shown in the Additional file, which shows that the TransCBCT can improve the accuracy of CT numbers and reduce the noise.

### 3.2 Clinical application value

**Figure 5:**
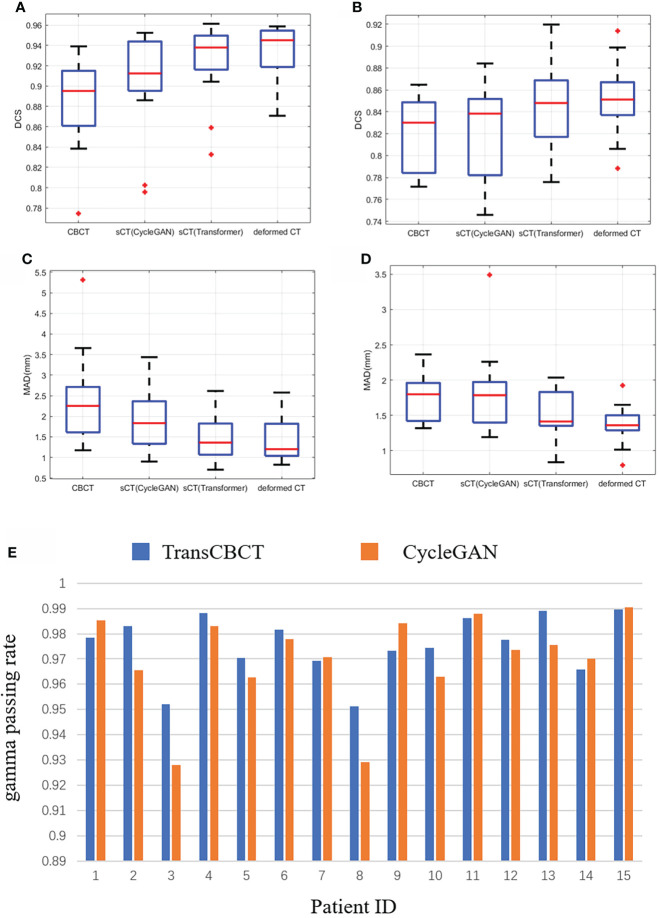
Statistical analysis of clinical value based on ART need. **(A–E)** Result of auto-segmentation. **(A)** DSC of bladder. The *p*-values were <0.05 (CBCT vs. deformed CT), <0.05 [sCT(CycleGAN) vs. deformed CT], and 0.196 [sCT(TransCBCT) vs. deformed CT]. **(B)** DSC of rectum. The *p*-values were <0.05 (CBCT vs. deformed CT), <0.05 [sCT(CycleGAN) vs. deformed CT], and 0.144 [sCT(TransCBCT) vs. deformed CT]. **(C)** MAD of bladder. The *p*-values were <0.05 (CBCT vs. deformed CT), <0.05 [sCT(CycleGAN) vs. deformed CT], and 0.450 [sCT(TransCBCT) vs. deformed CT]. **(D)** MAD of rectum. The *p*-values were <0.05 (CBCT vs. deformed CT), <0.05 [sCT(CycleGAN) vs. deformed CT], and 0.058 [sCT(TransCBCT) vs. deformed CT]. **(E)** Result of gamma passing rate for 15 patients. The results of TransCBCT were more robust compared to the CycleGAN. Some of CycleGAN results were under 93%.

We calculated the DSC and MDA between the predicted contour and ground truth. It is worth noting that the deformed CT and CBCT were contoured independently. Due to different scan times, the bladder and rectum on the images get different locals and sizes. For CBCT, sCT(CycleGAN), sCT(TransCBCT), and deformed CT, the mean of the DSC was 0.88, 0.90, 0.92, and 0.93 for the bladder and 0.82, 0.83, 0.84, and 0.85 for the rectum, respectively, and the mean of the MAD was 2.36, 1.92, 1.48, and 1.46 mm for the bladder and 1.75, 1.80, 1.52, and 1.37 mm for the rectum, respectively. As shown in **Figures 5A–D**, the result of TransCBCT was better than CycleGAN, and closer to the deformed CT (*p* > 0.05). The same auto-segmentation model was applied to these images, so the above result demonstrated that the image features of sCT(TransCBCT) and the deformed CT were more similar. In other words, TransCBCT improved CBCT to the level of deformed CT.

**Figure 5E** shows the result of gamma passing rate for 15 patients. The gamma passing rate of TransCBCT (1%/1 mm criterion) was 97.5% ± 1.1%. Compared to the result of 96.9% ± 1.8% for CycleGAN, there was a slight improvement, and the *p*-value was below 0.05. All the results of TransCBCT were above 95%. Even though the CycleGAN was in good performance, the TransCBCT showed more accuracy and robustness, which import to the ART. It is the result of less artifacts and more accuracy CT numbers on sCT(TransCBCT).

## 4 Discussion

The experimental results demonstrated that the proposed TransCBCT can effectively improve the quality of CBCT images. It can also preserve the structure of CBCT and calibrate the HUs effectively. The proposed TransCBCT is more suitable for ART due to its good performance in reducing the noise and artifacts than conventional CycleGAN. To the best of our knowledge, applying a transformer-based network for generating sCT has not been investigated yet.

The primary motivation of using a transformer-based network is due to its strength in capturing the long-distance dependence to the global. To be more specific, after calculating self-attention, one token is strongly related to other tokens. In comparison, the convolution-based network normally adopts a 3 × 3 or 7 × 7 convolutional kernel to capture the local context, and one pixel in the feature maps corresponds to a 3 × 3 or 7 × 7 field. To obtain long-distance relationship, the convolution-based network needs to be deeper, followed by other problems, such as diminishing gradient. There are also many advantages of convolution, such as low computational cost and translation invariance. The high computational cost also limits the application of the transformers on medical images. The proposed TransCBCT combined the advantages of convolution and transformer. The non-overlapping window design can reduce the model parameters to make it more effective. The inductive bias, which kept certain translation invariance, was still preferable for modeling. The network was trained on 6,144 paired images without pre-training. For one patient, the sCT images can be generated within 15 s. The computational cost was acceptable. To retain the advantage of capturing global information, hierarchical structure and SW-MSA were implemented. We also adopted several convolutional layers to enhance the ability to leverage the local context. In total, the network combines the power of the transformer in capturing the long-range dependencies and the advantage of convolution in leveraging the local context. The hierarchical encoder–decoder structure makes the network efficient to extract features and recover the image structures and details. The non-overlapping window-based and shift window-based strategies are essential in reducing the computational cost while maintaining efficiency.

**Figure 6:**
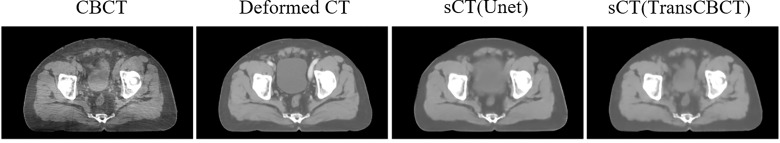
Result of a pure convolution-based Unet that made the shape of the bladder vary from CBCT. TransCBCT and Unet employed the same U-shape structure and loss function. However, with transformer block embedded, TransCBCT showed the ability to keep the raw CBCT structure.

The encoder–decoder structure with skip connection has been widely used in conventional pure convolution-based networks. U-net (34) is a typical and successful image processing network. Many studies (14–16) employed it to generate sCT from CBCT. However, when there are substantial structure changes between CBCT and deformed CT, the convolution-based Unet can easily be misled to a wrong optimized direction. **Figure 6** shows that pure convolution-based Unet cannot preserve the structure of CBCT. Meanwhile, the proposed TransCBCT also constructed the U-shape network; importing the transformer block helped to maintain the same structure as CBCT. This is important for ART since we want to obtain accurate online anatomical information from the images. Therefore, CycleGAN is the most popular network to generate sCT for ART. The result of CycleGAN shows that it can preserve the structure of CBCT and keep artifacts. The proposed TransCBCT can effectively reduce artifacts benefitted by leveraging the global information. TransCBCT shows more robustness and accuracy in calibrating the CT numbers.

The segmentation task is an important part of ART workflow, which affects the efficiency and accuracy. At present, many studies are focused on this task and contribute many efficient methods. For now, Yang Lei et al. (35) presented the best pelvic multi-organ segmentation result on CBCT images. They employed the CycleGAN to generate the MRI and then auto-segmentation is implemented on the synthetic MRI. Our center also employs the deep learning-based method to assist daily contouring work. The sCT (TransCBCT) showed more accuracy than sCT (CycleGAN) when using our trained auto-segment model before. Since the deformed CT is not well-matched to the CBCT, it is better to use the contour of CBCT as the ground truth of sCT. As shown in **Figure 4**, the shape of the bladder is different between the CBCT and the deformed CT. Thus, we contoured the organs on the CBCT and the deformed CT independently as the ground truth. The result of TransCBCT was close to the deformed CT, which means the features of sCT (TransCBCT) were closer to the deformed CT in image feature. The promising result might get a more long-term meaning on the radiomics reproducibility studies (36), which is helpful for ART clinical decision support. It is worth noting that the auto-segment model is a relatively objective evaluation standard, rather than a specific-constructed model for ART. However, since the sCT (TransCBCT) is close to the deformed CT, the problems on the segmentation part of the ART workflow would be easier to solve. In the future, a special deep learning network can be trained for ART workflow to improve accuracy. Our group performs relative studies on magnetic resonance imaging-guided adaptive radiotherapy (37).

As for dose calculation, even though the deformed CT cannot be regarded as the “Gold Standard”, there was no better reference for this study at present. Other studies (21, 23, 31, 32) also employed the deformed CT as reference for dosimetry evaluation for sCT generation from CBCT or MR. Uh et al. (23) used a CycleGAN, resulting in gamma passing rates of 98.5 ± 1.9% (2%/2 mm criterion) for proton dose calculation. Kurz et al. (38) obtained a result of 96% (2%/2 mm criterion) for the proton dose calculation and 89% (1%/1 mm criterion) for VMAT. We used a relatively well-performing CycleGAN and obtained a result of 96.9% ± 1.8% (1%/1 mm criterion) for photon VMAT, and the TransCBCT product showed superior results.

Based on our promising findings, the proposed framework has the potential to be applied in other image generation tasks, such as CBCT to relative stopping power maps and MVCT to sCT. The main contribution of CycleGAN is the design of cycle-consistent loss, which is important to keep the raw image structure. Our proposed network can also take advantage of the cycle-consistent loss by designing the cycle structure, which means that at least two transformer-based networks need to be employed as generators for the GAN model. The main challenge is how to deal with the high computing cost and how to balance the generator and discriminator.

## 5 Conclusion

In this study, we successfully developed a more effective CT synthesizer using transformers for CBCT-guided adaptive radiotherapy. The strength of the proposed method was also verified relative to the conventional pure convolution-based network. The sCT generated by TransCBCT is helpful for contouring and dose calculation, which can be used in ART to improve radiotherapy accuracy.

## Data availability statement

The datasets presented in this article are not readily available because of data security requirement of our hospital. Requests to access the datasets should be directed to KM, menkuo126@126.com.

## Ethics statement

The studies involving human participants were reviewed and approved by the Independent Ethics Committee of Cancer Hospital, Chinese Academy of Medical Sciences. Written informed consent for participation was not required for this study in accordance with the national legislation and the institutional requirements.

## Author contributions

All authors discussed and conceived the study design. XC wrote the programs and drafted the manuscript. YXL, BY, JZ, SY, and XX helped to collect the data and performed data analysis. YXL performed the clinical analysis. KM and JD guided the study and participated in discussions and preparation of the manuscript. All authors read, discussed, and approved the final manuscript.

## Funding

This work was supported by the National Natural Science Foundation of China (12175312), Beijing Nova Program (Z201100006820058), and the CAMS Innovation Fund for Medical Sciences (2020-I2M-C&T-B-073, 2021-I2M-C&T-A-016).

## Conflict of interest

The authors declare that the research was conducted in the absence of any commercial or financial relationships that could be construed as a potential conflict of interest.

## Publisher’s note

All claims expressed in this article are solely those of the authors and do not necessarily represent those of their affiliated organizations, or those of the publisher, the editors and the reviewers. Any product that may be evaluated in this article, or claim that may be made by its manufacturer, is not guaranteed or endorsed by the publisher.

## Abbreviations

CBCT, cone-beam computed tomography; ART, adaptive radiotherapy; sCT, synthetic CT; CycleGAN, cycle-consistent adversarial network; CNN, convolutional neural network; GAN, generative adversarial network; MSA, multi-header self-attention; LeFF, locally enhanced feedforward network; W-MSA, window-based MSA; MS-SSIM, multiscale structural similarity; MAE, mean absolute error; RMSE, root mean square error; PSNR, peak signal-to-noise ratio; ROIs, regions of interest; DSC, dice similarity coefficient; MDA, mean distance to agreement; VMAT, volumetric-modulated arc therapy.
