# Supplementary figures and images for: A more effective CT synthesizer using transformers for cone-beam CT-guided adaptive radiotherapy
Source: Front Oncol. 2022 Aug 25;12:988800. doi: 10.3389/fonc.2022.988800 (PMC9454309; doi:10.3389/fonc.2022.988800)

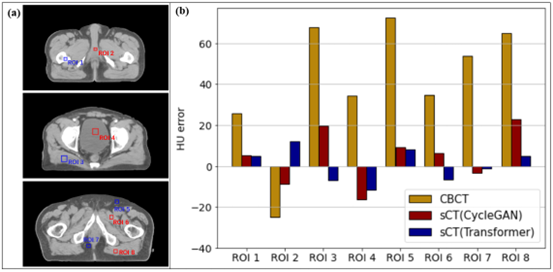

Supplement: Supplementary file 1 [file Image_1.png]
