# Supplementary material for: A more effective CT synthesizer using transformers for cone-beam CT-guided adaptive radiotherapy
Source: Front Oncol. 2022 Aug 25;12:988800. doi: 10.3389/fonc.2022.988800 (PMC9454309; doi:10.3389/fonc.2022.988800)
Supplement: Supplementary file 2 [file Table_1.docx]

The accuracy of CT numbers is import for the sCT. Eight ROIs (positions shown in Figure 1a) were selected to compare with the deformed CT. The average CT numbers on the deformed CT images in these ROIs were 317.0, 85.5, 48.7, 16.2, −74.5, 67.6, −79.2, and 63.3 HU, respectively. Compared to CycleGAN, the proposed TransCBCT improved the accuracy by 6.2%, 23.0%, 54.7%, 29.6%, 8.7%, 26.6%, 0.9%, and 22.1% for the eight ROIs, respectively. The proposed TransCBCT slightly outperformed the CycleGAN on HU calibration. The noise level was defined as the standard deviation of CT numbers in the ROIs. In addition, Table 1 presents the noise level in the eight ROIs, where the proposed TransCBCT performed better than the CycleGAN, with an improvement of 37.0%. The noise level was defined as the standard deviation of the CT numbers in the ROI. Overall, the proposed TransCBCT can effectively calibrate the CT numbers and reduce the noise.

**Figure 1.** (a) Three axial slices of the deformed CT with eight ROIs selected. (b) HU errors for eight ROIs chosen from (a) using deformed CT as a reference.

**Table 1**. Noise analysis of selected regions from Figure 1. for CBCT and sCT.

| ROI | 1 | 2 | 3 | 4 | 5 | 6 | 7 | 8 |
| --- | --- | --- | --- | --- | --- | --- | --- | --- |
| CBCT | 139.6 | 13.8 | 27.1 | 24.8 | 26.7 | 20.2 | 35.6 | 27.9 |
| sCT  (CycleGAN) | 108.1 | 6.3 | 11.6 | 12.0 | 10.2 | 6.2 | 8.3 | 7.9 |
| sCT  (TransCBCT) | 114.5 | 1.9 | 6.3 | 3.7 | 10.1 | 2.7 | 4.1 | 7.2 |
